# Dorsolateral prefrontal cortex-based control with an implanted brain–computer interface

**DOI:** 10.1038/s41598-020-71774-5

**Published:** 2020-09-22

**Authors:** Sacha Leinders, Mariska J. Vansteensel, Mariana P. Branco, Zac V. Freudenburg, Elmar G. M. Pels, Benny Van der Vijgh, Martine J. E. Van Zandvoort, Nicolas F. Ramsey, Erik J. Aarnoutse

**Affiliations:** 1 UMC Utrecht Brain Center, Department of Neurology and Neurosurgery, University Medical Center, P.O. Box 85500, 3508 GA Utrecht, The Netherlands; 2 Department of Neuropsychology, University Medical Center Utrecht, Utrecht, The Netherlands

**Keywords:** Neurology, Translational research

## Abstract

The objective of this study was to test the feasibility of using the dorsolateral prefrontal cortex as a signal source for brain–computer interface control in people with severe motor impairment. We implanted two individuals with locked-in syndrome with a chronic brain–computer interface designed to restore independent communication. The implanted system (Utrecht NeuroProsthesis) included electrode strips placed subdurally over the dorsolateral prefrontal cortex. In both participants, counting backwards activated the dorsolateral prefrontal cortex consistently over the course of 47 and 22 months, respectively. Moreover, both participants were able to use this signal to control a cursor in one dimension, with average accuracy scores of 78 ± 9% (standard deviation) and 71 ± 11% (chance level: 50%), respectively. Brain–computer interface control based on dorsolateral prefrontal cortex activity is feasible in people with locked-in syndrome and may become of relevance for those unable to use sensorimotor signals for control.

## Introduction

Locked-in syndrome (*LIS*) is characterized by intact cognition and (almost) complete loss of voluntary movement^1^. Brain–computer interface (*BCI*) systems allow computer control with brain activity and can therefore provide an alternative communication channel to people with LIS. BCI systems depending on self-initiated modulation of brain activity generally measure from sensorimotor areas, e.g.^2–9^. However, not all people with LIS may be able to reliably modulate sensorimotor activity, due to for instance atrophy secondary to neurodegenerative disease^10,11^, damage after stroke or injury, or atypical neural activity^12^. Therefore, it is important to investigate alternative signal sources for BCI-control.

One candidate source is the dorsolateral prefrontal cortex (*dlPFC*), which is activated by self-paced tasks such as mental arithmetic or serial subtraction^13–15^. Moreover, its signals can be measured from the cortical surface using electrocorticography (ECoG)^14,15^. We have previously shown that epilepsy patients with temporary ECoG implants can modulate high frequency band (HFB) power in the left-dlPFC by serial subtraction, and are able use these signals for one-dimensional cursor control^15^.

Here, we investigated whether people with LIS can use dlPFC signals and serial subtraction for reliable BCI control. Two people with LIS, equipped with a fully and chronically implanted BCI system, including electrodes over the left dlPFC, participated. To examine feasibility of dlPFC-based BCI-control, we investigated whether the participants could activate the dlPFC, whether they could use dlPFC activity for simple cursor control, and whether the neural features used for BCI control were stable.

## Materials and methods

### Participants

UNP1 was a woman diagnosed with ALS in 2008. She received invasive ventilation for the duration of this study. At the time of informed consent (September 2015) she was 58 years old. For communication she used an eye tracker to type, and eye blinks and (later) small movements of the mouth corner to answer closed questions. Besides eye movements/blinks and minimal facial movements, she was completely paralyzed (ALS functional rating scale score of 2 at time of informed consent).

UNP4 was a woman who suffered a brainstem stroke in 2004, limiting her motor capabilities to some neck and facial movements. She was 39 years old at the time of informed consent (August 2017). She used a head switch to control scanning software, and horizontal and vertical head and eye movements for answering closed questions.

### Assessment of mental calculation abilities

A neuropsychologist assessed the calculation abilities of the participants (detailed in^2^). Based on this assessment, an appropriate difficulty for research tasks was selected (medium/difficult for UNP1; easy for UNP4). We chose challenging arithmetic, because in an earlier study we found evidence for more prolonged HFB power increases with challenging arithmetic^14^, which is helpful in a task such as the target task (described below), where activity needs to be high for several seconds to hit the upper target.

### Localization of target area with fMRI

Localizing target regions prior to implantation is essential, given that there are interindividual differences in the cortical architecture of the dlPFC^14,16^. Therefore, functional magnetic resonance imaging (fMRI; 3 T, Philips Achieva) was used several weeks before implantation to determine locations for electrode placement. A PRESTO scan was used to reduce interference from large blood vessels^17^. Participants performed two tasks known to activate the left dlPFC (described below). Instructions were presented on a screen visible via a mirror attached to the head coil. Vital signs were continuously monitored by an anesthesiology team. UNP1 was ventilated by an MRI compatible ventilation machine. Researchers observed eyes of the participant via a camera focused on the eyes (UNP1) or via the head coil mirror (UNP4). Participants used eye blinks to answer yes–no questions and were instructed to blink continuously if they needed attention.

### fMRI mental calculation task

The mental calculation task consisted of 90 (UNP001, two runs) or 45 (UNP004, one run) equations: additions, subtractions, or a combination of both, plus an answer (e.g. 41–7 = 34). Candidates had to blink twice after deciding if the presented answer was correct, after which the trial was ended by a button press by a researcher. Maximum trial time was 10 s, after which trials ended automatically. A button press or timeout marked the onset of the inter-trial interval (ITI), which lasted for 10 s after a timeout, and 10 s plus remaining trial time after a button press. Participants were instructed to focus on a fixation cross during the ITI. The period between trial start and end (by button press or timeout) was considered ‘active’, all else ‘rest’.

### fMRI count task

The fMRI count task consisted of three alternating blocks: rest, self-paced count back, and self-paced count forward. Each block lasted 15 s. During rest trials, the participant looked at a zero presented on the screen. During count back, the user repeatedly subtracted a step-size number from a starting number, both randomly generated and presented on the screen for the duration of an active block. During count forward, participants counted forward in steps of 1, starting from a random starting number between 1 and 10 (presented on screen). The fact that both participants were locked-in prohibited monitoring of counting performance during this task.

### fMRI data analysis

fMRI data analysis was performed in single subject space. Functional images were realigned and co-registered to T1-weighted anatomical images. We produced t-maps, contrasting count back (count task) or mental calculation (mental calculation task) with rest. Areas showing clear positive blood oxygen level dependent (*BOLD*) activity within dlPFC (based on^15^) during both tasks were selected for electrode placement.

### System implantation and data specifications

Details about the implanted components and surgical procedures have been described before^2^. Briefly, electrode strips (4 electrodes each; Resume II ®, Medtronic; off-label use) were implanted subdurally over the left sensorimotor hand area (electrode numbers e1 to e4) and left dlPFC (e8 to e11). During surgery, we used neuronavigation to place the electrode strips precisely over the target areas that were determined using presurgical fMRI. Each strip was inserted between the dura and pia mater, going from one burr hole to a second, and was attached to the skull at both ends, thus making sure strips were placed according to the surgical plan. In this paper, only results from the dlPFC electrodes are discussed. Leads were tunneled behind the left ear and connected to an amplifier/transmitter (Activa ® PC+s, Medtronic; off-label use) implanted under the left clavicle.

Bipolar referencing was used for all data recordings. The amplifier can stream raw voltage data at 200 Hz (time-domain data) for one bipolar electrode pair from each strip. It can also convert raw potential into power-data using on-board chopper-based band pass filtering, which is streamed at 5 Hz and allows for transmission of power-domain data from three bipolar channels when measuring from one strip.

### Research tasks

During home visits, participants routinely performed two working memory tasks. Research tasks were developed in-house, based on BCI2000 software^18^.

#### Count task design and statistical analysis

The goals of the count task were: (1) to obtain the optimal bipolar electrode pair and select an appropriate frequency band for control; (2) to follow the stability of the neural features used for control. This count task was similar to the fMRI count task, but only had two conditions: count back and rest (15 s each). During count back, participants were visually cued with numbers to be used for serial subtraction. UNP1 was presented with randomly generated starting and step size numbers (e.g. 417–23) on each trial. UNP4 was only presented with randomly generated starting numbers (e.g. 88), because she always used the same step size number within one run. These parameters were based on the mental arithmetic level of the participants, as assessed by a neuropsychologist before electrode implantation, and were chosen to be challenging, but not too difficult. During rest blocks, they looked at a zero in the middle of the screen. Task duration for UNP1 was 5 min during the first 10 weeks and 2 min thereafter. Task duration was always 3 min for UNP4.

Count task data were recorded in time-domain (200 Hz; high pass filter 0.5 Hz). For analysis, data was converted to power-domain using an autoregressive filter (bin range 1–99 Hz; bin width 1 Hz; model order 20; window length 200 ms; 5 evaluations per bin, equally spaced at 0.2 Hz, which are averaged to get the power for one 1 Hz bin). Power of each 1 Hz bin was averaged for each active and rest block, resulting in one power value per bin for every 15 s rest and active block. These values were then correlated with a binary sequence of zeroes and ones (indicating rest and active trials, respectively), to obtain a correlation and *p* value for each 1 Hz bin. For example, if a run included six rest and six active trials, we correlated an array of twelve power values with a binary array of the same length. The resulting correlation value was squared to calculate the signed coefficient of determination (r^2^). Using power data averaged across active and rest blocks, spectrograms (range: 1–99 Hz) were plotted for both conditions, plus the signed r^2^ values from all bins and associated significance level (based on uncorrected *p* values and α = 0.05). Also, for each active and rest block, the average HFB power (65–95 Hz, previously used for the same paradigm^15^) was calculated by averaging the 65–95 Hz bins. r^2^ and *p* values were then calculated in the same way as described above. We report r^2^ values and corresponding statistical significance (based on uncorrected *p* values and α = 0.05). A linear regression model was used to check for a trend in r^2^ values.

Approximately every 12 (UNP1) or 6 weeks (UNP4), we tested all 6 bipolar electrode combinations in one recording session. Each combination was tested in a separate run, because the amplifier streams time-data from one electrode pair from each strip at a time. In the first year after implantation, data from the optimal bipolar pair was recorded more frequently to measure signal stability (approximately every 2 weeks; some periods have missing data due to vacation breaks). Electrode pair e8–e10 turned out optimal for both participants, based on initial tests of all pairs and electrode location with respect to fMRI hotspot. After the first year, research session frequency of UNP1 was decreased, explaining the sparsity of data in that period.

#### Target task design and statistical analysis

The goals of the target task were to investigate whether participants could control a BCI task with activity from dlPFC and to measure stability of performance. Participants used HFB power from one bipolar electrode pair of the dlPFC strip to control y-dimension velocity of a cursor to hit a target (up or down) on the right side of the screen. The cursor moved from left to right at a fixed pace, and participants were instructed to send the cursor up or down by performing serial subtraction or rest, respectively. For UNP1, either both a starting number and step size were presented on the left side of the screen at the onset of each trial, or—between weeks 12 and 31 and after week 48—no numbers were presented (no cues), meaning she used self-generated starting numbers (and fixed step size numbers). For UNP4, a starting number was presented, and she used the same step size throughout a run (first session: 2; then: 3; after 25 weeks: 6). She did 6 runs without cues.

To keep the task challenging and engaging, task parameters were varied, based on performance and user feedback. Trial length was decreased from 6 to 2 s for UNP1 and from 6 to 4 s for UNP4. Score (percentage of correct trials) was presented after each run. During the first run of each session, adaptation was used to correct for potential fluctuations in brain signal and to properly scale data to the y-dimension. If the participant reported good control, settings were fixed in the following run(s); if not, another adaption run was done. The length of the moving adaptation window was arbitrarily set to 9 s, meaning neurofeedback would only start after an initial 9 s of feedback time had been recorded. Therefore, trials taking place before 9 s of feedback time had passed, were excluded from the score calculation used for this paper. Random re-shuffles of task events on real data 10,000 times revealed a chance score of 50% (± standard deviation of 7% and 11%, respectively for runs with the largest {50} and smallest {20} number of trials recorded in one run). A linear regression model was used to check for a trend in performance.

Run duration was 2 or 3 min (adaptation runs) and 5 min (fixed settings) for UNP1. Runs of UNP4 always lasted 3 min. Most target task data were recorded in power domain (center frequency between 50 and 80 Hz; bandwidth 2.5 Hz; high pass filter 0.5 Hz). In the few time-domain runs, data was converted on the research computer into power-domain using an autoregressive filter (bin width: 1 Hz; model order: 20; window length 200 ms; evaluations per bin: 5; control signal based on HFB power with a center frequency between 50 and 80 Hz). Both participants always used electrode pair e8–e10 for control.

### Ethical approval and informed consent

The medical ethics committee of the UMC Utrecht approved this study (Utrecht NeuroProsthesis; ClinicalTrials.gov: NCT02224469, registration date: 25/08/2014) which was carried out in accordance with the Declaration of Helsinki (2013). Participants gave informed consent through a procedure dedicated to people with LIS (described in^2^).

## Results

### fMRI and electrode strip placement

**Figure 1:**
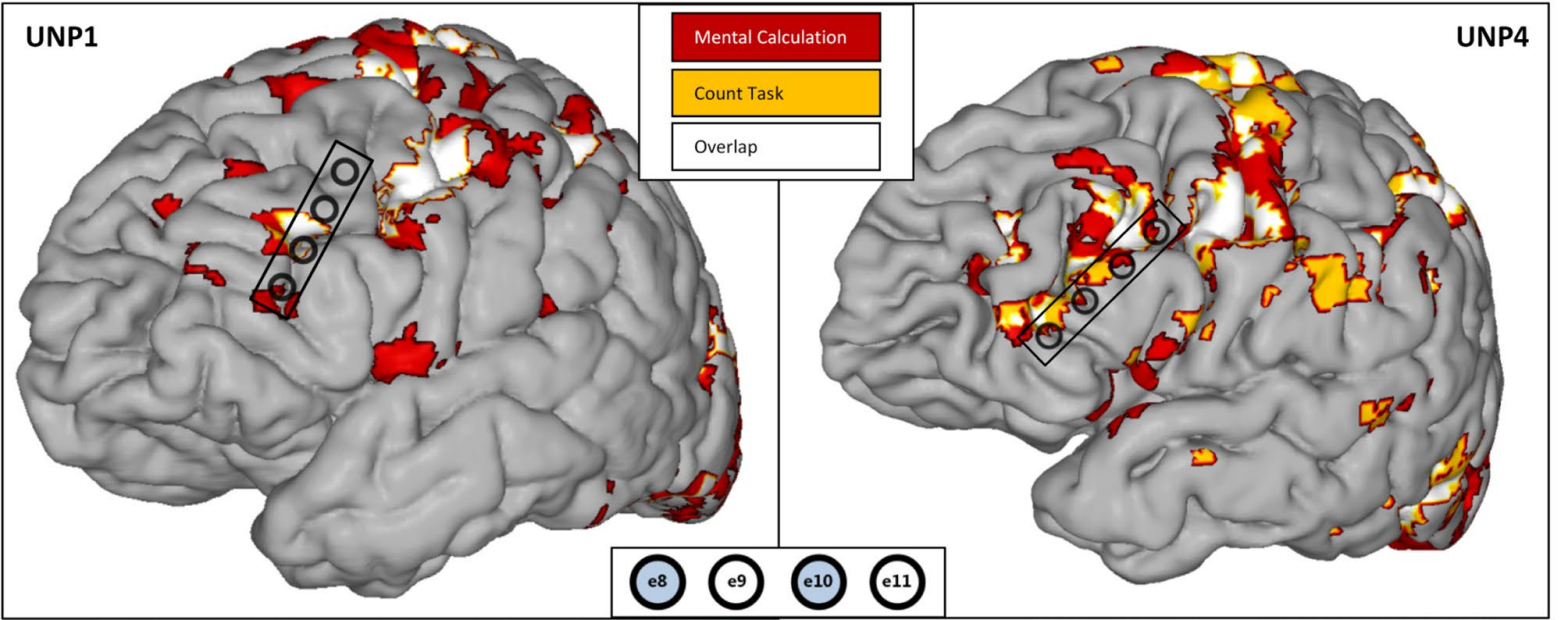
fMRI results and electrode strip location. T-maps from the mental calculation task (red), count task (orange), and their functional overlap (white) from both participants (left: UNP1, right: UNP4) plotted on their anatomical T1 weighted images (method from^21^, supplementary materials). Applied thresholds: UNP1: t > 10 for both tasks; UNP4: t > 7 and t > 3 for mental calculation and count task, respectively; voxel depth 8 mm. Strip and electrodes placed over the left-dlPFC are indicated in black on the brain surface. Electrode numbers are indicated at the bottom of the figure, e8 being the most frontal and inferior electrode in both participants. Electrodes used for control in both participants are indicated in light blue. In our visualization software, different colors are represented numerically (1–3 for mental calculation task, count task, and the overlap, respectively) and edge colors take one value lower: hence the different edge colors for different activation maps in these plots.

fMRI analysis revealed increased activity during active blocks of the count and mental calculation tasks in the left dlPFC area of both participants (Fig. 1).

### Count task

**Figure 2:**
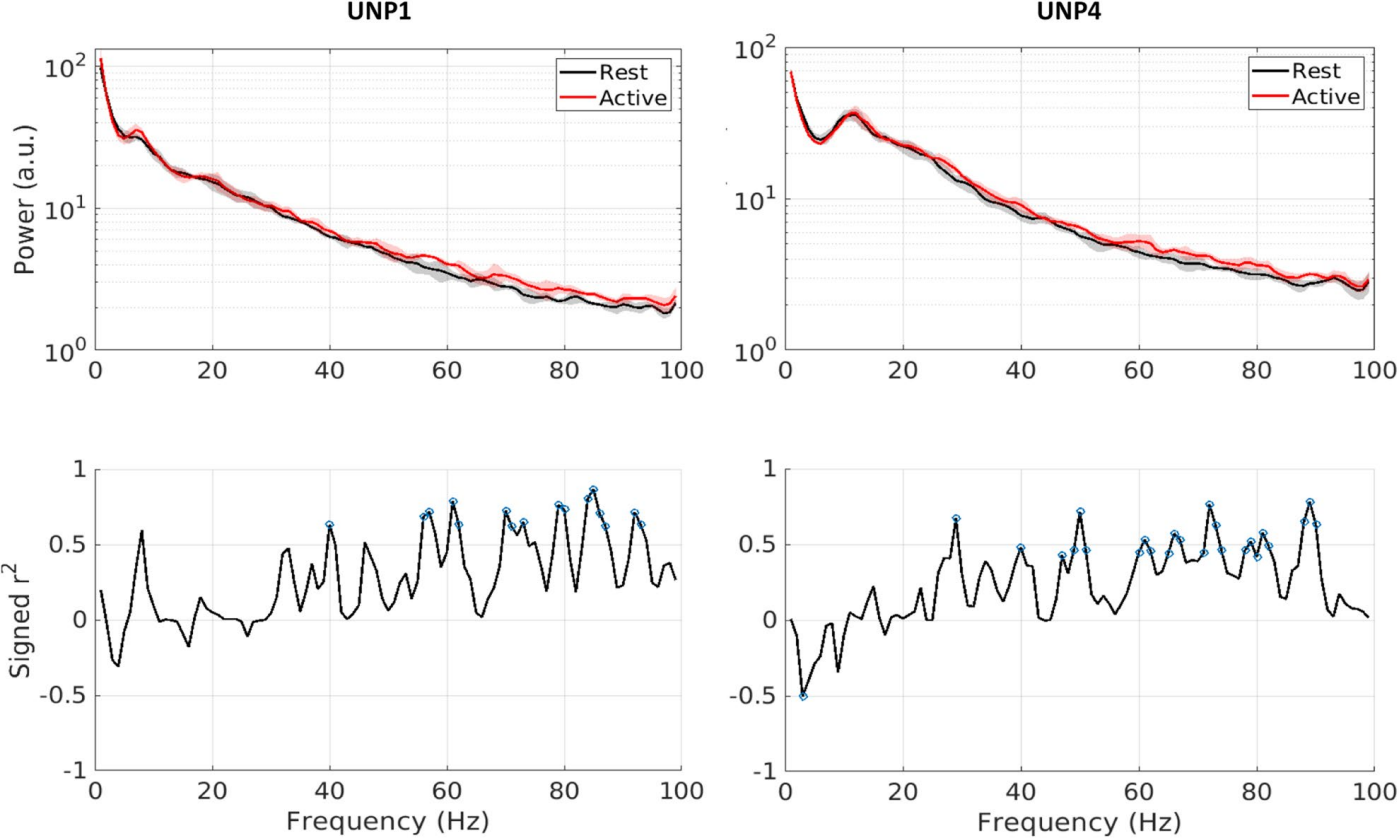
Representative examples of spectral power changes during the count task. Top graphs show a spectrogram of both conditions for a representative run from each participant (red is active, black is rest). Bottom graphs show respective signed r^2^ values for each 1 Hz bin, with blue circles indicating significant r^2^ values (α = 0.05; *p* values not corrected). Plotted data is from week 9 (UNP1) and week 22 (UNP4). Both runs were recorded with pair e8–e10.

Power spectra were produced for each run and generally showed increased HFB power during active blocks (representative power spectra from both participants are plotted in Fig. 2).

**Figure 3:**
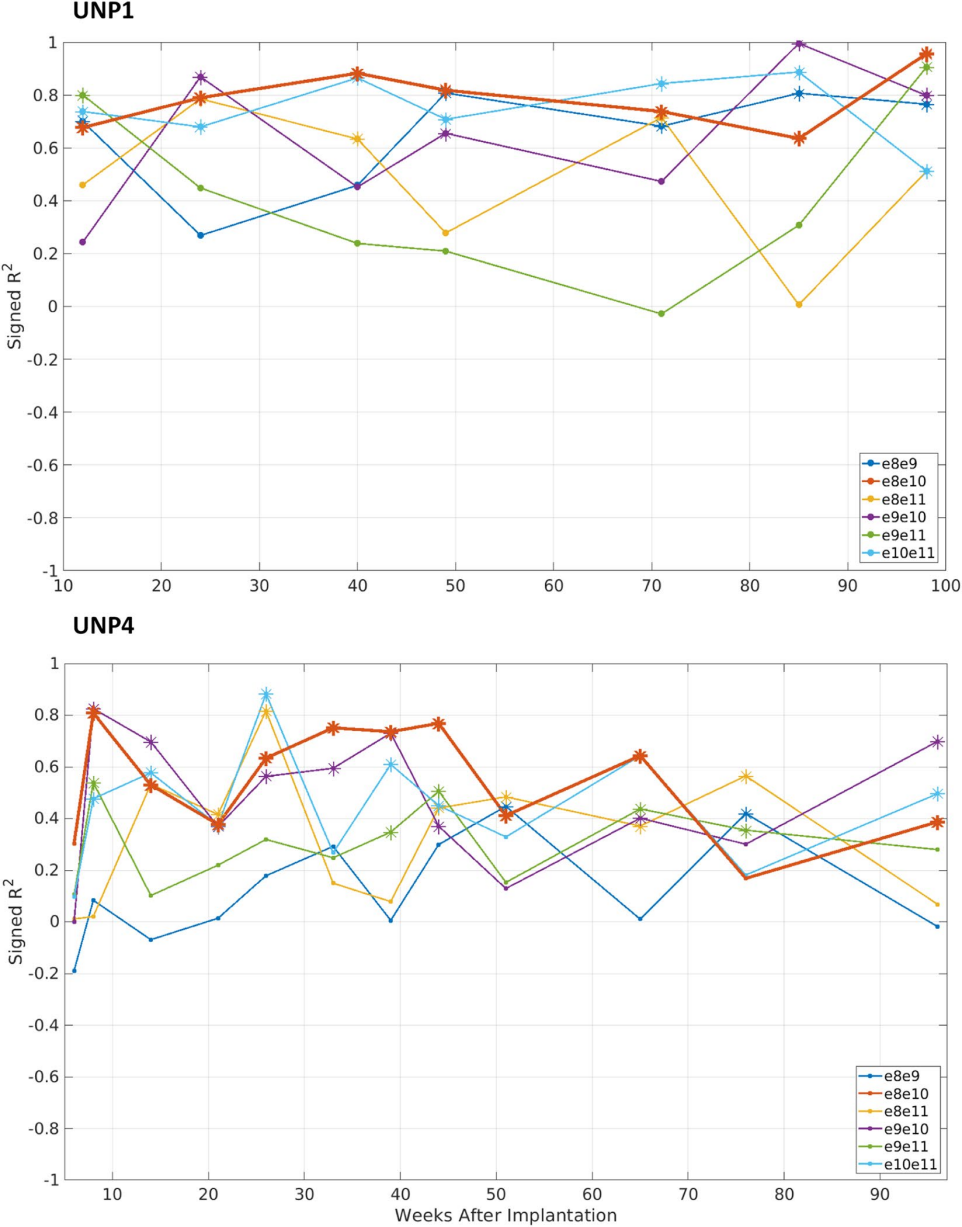
Signed r^2^ values from all bipolar electrode combinations. r^2^ values between HFB power (65–95 Hz) and task conditions, from all bipolar pairs, with weeks since implantation on the x-axis. Different lines indicate different bipolar pairs (see legend), and asterisks indicate significant HFB activation during active blocks (α = 0.05; *p* values not corrected).

Evaluation of r^2^ values for all bipolar electrode pairs (only data from sessions in which all pairs were recorded) revealed that in both participants the count task induced a clear increase in HFB (65–95 Hz) power in one or more electrode pairs, as indicated by high and significant r^2^ values (Fig. 3). For UNP1, two pairs (e8–e10 and e10–e11) showed a significant r^2^ value in all sessions. For UNP4, no significant r^2^ value was found in the first session (the only session in which she subtracted 2 from the starting number; she increased the step size number in subsequent sessions). Moreover, in the penultimate session of UNP4 the pair e8–e10 did not show a significant HFB power effect. Pair e8–e10 was significant in all other sessions.

**Figure 4:**
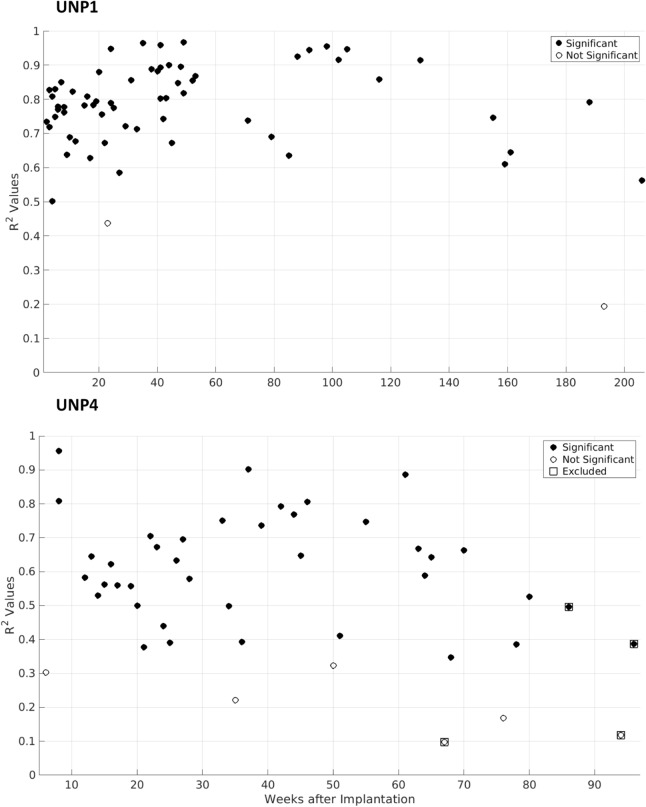
Signed r^2^ values from control pair e8–e10. For UNP1 (top) and UNP4 (bottom), the signed r^2^ values between HFB power (65–95 Hz) and count task conditions from all runs with pair e8–e10 are plotted, with weeks since implantation on the x-axis. Y-axis runs from 0 to 1, because no r^2^ value from pair e8–e10 fell below 0. Black dots indicate significant HFB activation during active blocks (α = 0.05; *p* values not corrected). Because task duration was varied and *p* values are influenced by sample size (i.e. the number of blocks), the statistical significance of specific r^2^ values may vary (e.g. UNP1′s 0.44 in week 23 is not significant, even though UNP4′s 0.39 in week 21 is). Runs from UNP4 recorded during high fatigue are indicated by squares and were removed from final linear regression. Values from electrode pair e8–e10 from Fig. 3 are included in this figure.

Electrode pair e8–e10 was recorded more frequently. UNP1 and UNP4 performed 64 and 45 count task runs across a period of 206 and 96 weeks, respectively, while neural signals from this pair were recorded. Average r^2^ values for UNP1 and UNP4 were 0.78 ± 0.14 (mean and standard deviation) and 0.56 ± 0.21 (Fig. 4). In the run of week 159 UNP1 had her eyes closed and was cued verbally by the researcher and in week 193 no cues were provided. In all other runs from both participants the instructions were as described in the methods section. Linear regression revealed no trend in r^2^ values for UNP1 (*p* > 0.05) and a downward trend for UNP4 (*p* = 0.03). Notably, during several sessions, UNP4 was experiencing high fatigue (mostly in the latter part of the study, when task frequency was consequently lower). When the respective runs (weeks 67, 86, 94, and 96; squares in Fig. 4) were excluded from the linear regression, no trend in r^2^ values was present (*p* = 0.4).

### Target task

**Figure 5:**
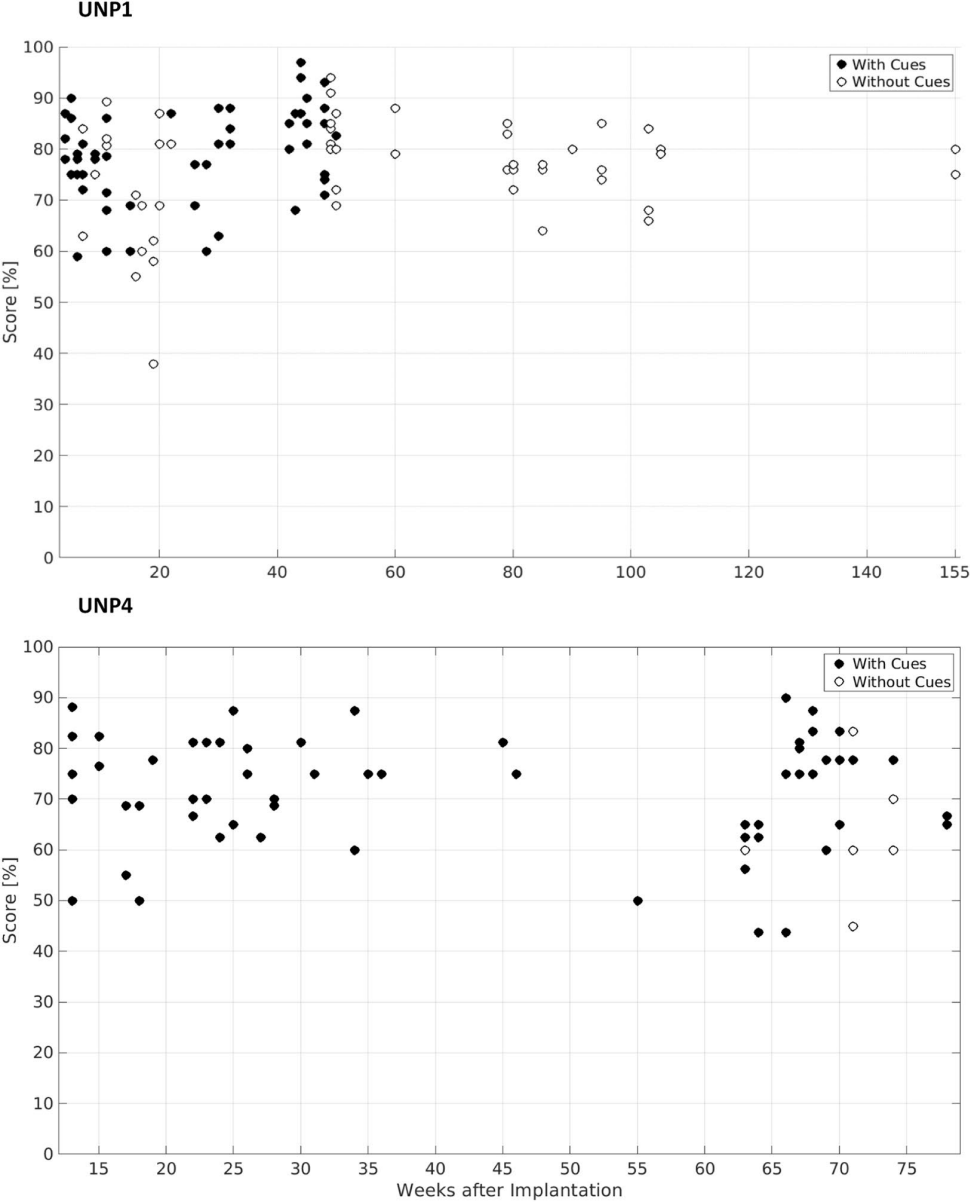
Target task performance. Performance on the target task for both participants, with weeks since implantation on the x-axis, and score (0–100%) on the y axis. Chance level is 50%. Fill indicates strategy (black means with cues; white without). Because runs with the same score from one session are plotted on the same location, the number of dots in these figures does not match number of reported runs.

UNP1 did 119 runs of the target task across 155 weeks (Fig. 5, top panel). Five runs were aborted, either due to technical problems (twice, weeks 45 and 50), per request of UNP1 due to poor BCI control (twice, week 90), or due to physical discomfort of UNP1 (week 50). Aborted runs are not plotted or used for data analysis. The average score across the remaining 114 runs was 78% ± 9% (mean ± SD). UNP1 performed the task with and without cues (67 and 52 runs, respectively) and switched to a no-cue strategy twice. In no-cue runs she chose a starting number for each trial, but used a fixed step size number. A two-sample *t* test comparing scores from runs with and without cues up to week 21 (after week 21 she switched back to *with* cues) shows that removing cues initially led to lower performance (*p* < 0.01). However, after switching to no cues a second time in week 48, scores did not differ between runs done *with* (from week 21 to 48) and *without* cues (week 48 and onwards; two sample *t* test, *p* = 0.19). Linear regression revealed no significant trend over time across all scores (*p* > 0.05).

UNP4 did 66 target task runs across a period of 78 weeks with an average score of 71% ± 11% (Fig. 5, bottom panel). None of her runs were aborted. Six of her runs were done without cues. Linear regression revealed no significant trend across all scores (*p* > 0.05).

## Discussion

Our results show that both locked-in participants could modulate HFB power in their left dlPFC by performing mental arithmetic and could use this for one-dimensional cursor control. They were able to activate the left dlPFC from the beginning of the study, indicating no training was required for willful modulation. HFB modulation was stable, as evidenced by the absence of a significant trend in count task r^2^ values and target task performance. Our data indicate that the dlPFC could be a promising alternative when the sensorimotor cortex cannot be used for BCI control.

Even though these results are promising and within the same range as those reported for three able-bodied epilepsy patients (average r^2^ values: 0.79, 0.53 & 0.65; mean and standard deviation of target task scores: 91 ± 2, 70 ± 8, & 74 ± 5%^15^), performance was not perfect: some count task runs showed no significant HFB power modulation, and target task scores were not always higher than chance level. Moreover, compared to sensorimotor results from the same implanted system, count task r^2^ values and target task scores presented here for UNP1 are lower (motor task r^2^: 0.89 ± 0.16; target task scores: 91 ± 6%; for details see^2^). This difference could be due to the nature of mental calculation, which may be less consistent than motor attempt. Indeed, we observed strong within and between session variation in r^2^ values and target task performance (e.g. count task from UNP1 in week 41 and target task from UNP4 in week 67). One possible explanation for this variation is that different trials may have different degrees of difficulty, depending on the numbers used in a specific trial. In studies using the same mental calculation task, increased difficulty led to a marked (several seconds) delay of HFB modulation onset in the dlPFC as measured with ECoG^14^ and a delayed BOLD response^13^. Even though we cannot properly check the effect of difficulty because it was relatively constant, small variability in difficulty may have been present. Also, work done in our group with epilepsy patients already showed inconsistent timing of dlPFC modulation^19^. Moreover, when too difficult, no mental calculation may take place (i.e. the participant does not try to solve the equation). Timing is especially relevant because controlling scanning-based communication software depends on correctly timed clicks. Exploring the effect of task difficulty and strategy on the timing and consistency of HFB modulation is important for boosting dlPFC-based BCI performance and deserves attention in future studies.

Training to activate the dlPFC without cues is important for eventual home use, since the control strategy should not depend on cues. After training, UNP1 could control a cursor without cues, by generating a starting number herself, effectively using a combination of random number generation and mental arithmetic, both of which have been associated with changes in dlPFC activity (random number generation^20–23^; mental arithmetic^13–15^). The fact that we did not observe training related changes in r^2^ values suggests that training in UNP1 affected mostly the mental strategy and her ability to control the BCI without cues, rather than the brain signals themselves. Due to fatigue (unrelated to the study), UNP4 performed fewer runs per session than UNP1. Subsequently, UNP4 performed fewer working memory tasks than UNP1 and only attempted the target task without cues six times. To achieve cue-independent BCI control with dlPFC, future work could focus on different arithmetic strategies and their influence on dlPFC activity.

Besides an alternative, dlPFC could be a valuable complement to sensorimotor BCIs. First, both participants reported that dlPFC-based BCI control was less tiring and required less mental effort than sensorimotor-based BCI control. We speculate that this may be related to the fact that the participants are still able to perform mental arithmetic (an everyday cognitive task), but can no longer actually move, which may therefore be associated with more mental effort and fatigue. Some users may prefer lower effort and fatigue at the cost of performance and speed. If future BCI systems measure from both regions, we envision users switching between dlPFC and sensorimotor control at will. Another reason in favor of dlPFC as a complement is the possibility that users may experience difficulty controlling sensorimotor-based BCIs in some situations, e.g. when being moved during care or transport. In these situations, sensory input and passive movement of body parts may inadvertently activate the sensorimotor cortex, making sensorimotor BCI control unreliable. A backup area that is not affected in these situations—like the dlPFC—may be useful. If this could allow a minimal degree of autonomous communication at all times (e.g. attracting attention of a caregiver), it would be a worthwhile addition to any communication BCI.

One limitation of this study is the small number of participants. How these results generalize is unknown. Furthermore, due to our focus on sensorimotor-based BCI, no systematic attempts to use dlPFC signals for control in click-based applications (e.g. spelling) have been made. A possible limitation of dlPFC-based BCI in general concerns the heterogeneity of the dlPFC and the interindividual differences in the precise location of the most suitable target areas for control. One approach to address this issue is to conduct a pre-surgical MRI scan to identify these locations in each individual. Yet, it cannot be excluded that, in daily life implementation of this approach, there may be interference from mental tasks that *inadvertently* activate the dlPFC, like processing of visual and auditory stimuli from extra-personal space^24^, verbal and spatial processing^25^, and spatial memory^26^. How these and other tasks interfere with dlPFC-based BCI control requires further research. Especially important is the effect of spelling-related activity on dlPFC-based BCI control.

From the first steps we have taken here with locked-in users of a dlPFC-based BCI, we conclude that the dlPFC could be an alternative or complementary brain area for BCI control. Necessary steps towards reliable home-use of a dlPFC-based BCI include improving consistency of dlPFC-modulation, training of self-generating dlPFC signal changes, click-based BCI applications with dlPFC activity, and elucidating how mental activity—including language—interferes with dlPFC-based BCI control.

## Data Availability

At the end of this study, data can be shared by the corresponding author upon reasonable request.
